# Prevalence and Determinants of Adult Under-Nutrition in Botswana

**DOI:** 10.1371/journal.pone.0102675

**Published:** 2014-07-23

**Authors:** Gobopamang Letamo, Kannan Navaneetham

**Affiliations:** Department of Population Studies, University of Botswana, Gaborone, Botswana; University of Malawi, Malawi

## Abstract

**Background:**

To estimate the prevalence and determinants of adult under-nutrition in Botswana.

**Methods:**

A cross-sectional survey was conducted where a nationally representative sample of people aged 20 to 49 years was used for the analysis. The outcome measure of under-nutrition was measured as BMI<18.5 kg/m^2^.

**Results:**

Of the total sample, 19.5% of males and 10.1% of females were underweight (BMI<18.5 kg/m^2^). The wealth index showed that 30.9% of the adult population with low a BMI belongs to the poorest 20% of the households while only 9.6% comprised of the richest 20% of the households. Results from logistic regression analysis indicated that both adult men and women who had no education and belonged to the low socioeconomic group had a statistically significant association with low BMI. Among the female adult population, being young and not having watched TV at least once a week were significantly associated with low BMI. For the male adult population, being unmarried was significantly associated with low BMI.

**Conclusions:**

Programme interventions aimed at improving the nutritional status of adults can use these findings to make appropriate policy, to establish baselines and study nutritional changes over time and its covariates.

## Introduction

The consequences of under-nutrition among adults and children are well known. Under-nutrition is a greater risk factor for low productivity, poor health and mortality [1]. Moreover, under-nutrition among women leads to poor reproductive health outcomes [2]. On the other, health professionals have warned about the adverse health outcomes of overweight and obesity [3]. While warnings about health consequences of excess weight abound [4]–[6] much less attention seems to be paid to the implications of being underweight [7]–[8]. This may partly be explained by the fact that underweight is not as prevalent as being overweight, particularly in the developed world. Another explanation emanates from the fact that thinness is commonly regarded as an ultimate goal to the extent that it is hard to think of thinness as a health concern [7]. As such there is limited research on the health and well-being of underweight adults in the context of Sub-Saharan Africa, particularly in Botswana. Both underweight and obesity are forms of under-nutrition and have greater health consequences. However, with respect to human development perspective, underweight demands as high degree of priority as it signifies the lack of food security than obesity which indicates over consumption of food.

Under-nutrition and poor health from preventable causes disproportionately affect the well-being of millions of people in the developing countries [9]. Fear of being fat may induce unnecessary attempts to reduce body weight [10], producing thinness that in some cases is associated with nutritional deficiencies, irregular menstruation, and eating disorders [11]–[13].

Previous studies suggest that underweight in women of childbearing age is a risk factor for adverse pregnancy outcomes, such as intrauterine growth retardation or low-birth weight infants [14]–[16]. Low BMI can be a sign of chronic energy deficiency (CED) and lack of adequate weight gain during pregnancy can lead to low birth weight babies leading to adverse health implications [17]–[18]. Women with CED have increased morbidity [18] while other studies have linked low BMI to decreased work capacity [19]–[21]. Among individuals who are HIV-positive, those with lower BMI may progress to AIDS more quickly [22]. It has been noted that information on the prevalence of under-nutrition in adults in developing countries is mainly restricted to data on women [23]. They further note that differences between under-nutrition prevalence in adult men and adult women might occur but systematic information on the subject is lacking. In developing countries like Botswana, estimates of the prevalence of underweight among the adults are lacking. This study attempts to fill this gap.

Countries in the world are experiencing increasing rates of overweight and obesity [24]. However, Sub-Saharan Africa (SSA) experiences both the burden of over-nutrition and under-nutrition [25]. A number of studies found that several African countries have high prevalence of overweight and obesity [26]. Literature on the prevalence of overweight and obesity in Africa and its associated factors is enormous. However study related to under-nutrition especially among the adult population is lacking.

The purpose of this study is to investigate the prevalence of adult under-nutrition among adult population in Botswana. Attempt is also made to identify the demographic, social and economic correlates of under-nutrition. And finally, the study examines the gender gap in under-nutrition and unravels how the correlates of under-nutrition vary among adult males and females.

## Methods

### Study Design and Participants

This study is a cross-sectional study conducted by Central Statistics Office (now Statistics Botswana). The Botswana Family Health Survey IV (BFHS IV) was conducted in September 2007–January 2008 by the Central Statistics Office. Four questionnaires were administered, namely the Household Questionnaire, Female Questionnaire (aged 12–49), Male Questionnaire (aged 12–49) and the Under 5 Questionnaire [27]. The survey collected information on adult weight and height for calculation of Body Mass Index (BMI).

### Data source and sample size

Respondents aged 20–49 years were selected for the analysis. Analysis for men and women were done separately. The sample consists of 4916 for females and 4107 for males. The sample was weighted to ensure that it is nationally representative.

### Outcome variable

Under-nutrition among adults is commonly assessed by using body mass index (BMI), defined as the weight in kilograms divided by the square of the height in metres (kg/m^2^). Under-nutrition or Chronic Energy Deficiency (CED) is usually defined when the BMI values is less than 18.5 kg/m^2^. Underweight is also divided into its constituent parts: severe thinness (BMI<16.0 kg/m^2^), moderate thinness (16.0< = BMI<16.99 kg/m^2^) and mild thinness (17.0< = BMI<18.49 kg/m^2^) [28]–[29]. BMI used in the logistic regression analysis was coded as 1 if the respondent is undernourished (BMI<18.5) and coded as 0 if not. The extreme values in the measurement of height and weight for both males and females have been excluded from the analysis. The analysis also excludes women who were pregnant at the time of the survey.

### Explanatory variables

The following explanatory variables have been considered for the analysis. Age in years was categorized as follows: 20–24, 25–29, 30–34, 35–39, 40–44 and 45–49. The place of residence was aggregated as city/town, urban village and rural. Marital status was created as never married, currently married (married and living together) and formerly married (separated, divorced and widowed). Educational level was measured as no education, primary, secondary and higher.

The BFHS did not collect household income or expenditure. However, the survey collected information on household assets or durables. Wealth index was constructed using this information applying the method proposed by Filmer and Prichett [30]. The household assets or durables included are: ownership of van/bakkie; car; tractor; donkey cart; bicycle; wheelbarrow; radio; TV; computer; refrigerator; watch; cellphone; landline; mokoro/canoe; boat with motor; sewing machine; motorbike; and plough. The weights for each of the assets were generated through Principal Components Analysis method. The wealth index has been computed for each household using the weights and households were classified into five wealth quintiles group.

### Statistical Methods

Data were analyzed using SPSS version 20. The prevalence of underweight across different categories was computed using cross-tabulations. The association between the dependent variable (whether the respondent was undernourished or not) and each of the independent variables were established using Pearson’s chi-square test. To identify the important covariates for under-nutrition, multivariate logistic regression analysis (1 = if a respondent is undernourished; 0 = otherwise) was applied and the estimated adjusted odds ratios (OR) gives relative importance of the covariates.

### Ethics Statement

The authors used secondary datasets obtained from the national statistical office, Central Statistics Office.

## Results

### Prevalence of under-nutrition

Table 1 presents prevalence and patterns of under-nutrition. It shows that 19.5% of males and 11.5% of females were undernourished (BMI<18.5 kg/m^2^). The mean BMI was higher among women (24.4±5.8 kg/m^2^) than men (21.7±4.7 kg/m^2^). Table 1 shows the prevalence of underweight by different types: severe thinness, moderate thinness and mild thinness. Underweight adult men and women who were mildly thin were 13.7% and 6.2%, respectively. About 4.1% of men and 2.7% of women were moderately thin. The proportion of adult men and women who were severely thin were similar, standing at 1.7% each. Thus underweight adult population was predominantly moderately thin. Low BMI was more pronounced among men than among women.

**Table 1:** Percentage of underweight and type of thinness among adults aged 20–49 years by sex, 2007 Botswana Family Health Survey IV.

Socio-demographic characteristic		Men (n = 4095)				Women (n = 4904)			
		Type of thinness			Normal & over-weight	Type of thinness			Normal & over-weight
		Severe Thinness	Moderate Thinness	Mild Thinness		Severe Thinness	Moderate Thinness	Mild Thinness	
Age	20–24	2.0	3.6	18.1	76.3	2.2	3.6	11.8	82.4
	25–29	1.5	4.5	13.7	80.3	1.7	4.0	7.4	86.9
	30–34	2.2	3.5	12.8	81.5	1.3	2.4	5.3	90.9
	35–39	1.2	4.1	11.5	83.2	1.0	1.3	5.3	92.5
	40–44	1.8	5.0	9.8	83.5	1.4	1.2	4.3	93.2
	45–49	1.0	4.5	10.1	84.4	2.5	0.7	3.5	93.3
		?^2^ = 34,4**				?^2^ = 101.1***			
Place of Residence	City/town	1.4	3.0	10.3	85.3	1.5	1.9	6.2	90.4
	Urban village	1.9	3.9	13.0	81.2	1.3	3.4	7.1	88.2
	Rural	1.9	5.1	17.4	75.6	2.2	2.6	6.2	87.1
		?^2^ = 44.2***				?^2^ = 15.7*			
Marital Status	Currentlymarried/in union	1.0	3.1	9.8	86.1	1.2	1.9	6.5	90.4
	Formerlymarried/in union	0.0	2.4	7.1	90.5	1.0	2.1	1.6	95.3
	Never married/in union	2.3	4.9	17.1	75.7	2.2	3.6	8.3	85.9
		?^2^ = 72.3***				?^2^ = 38.4**			
Educational Level	No education	1,6	8.1	14.4	75.9	4.3	3.3	10.3	82.1
	Primary	2.2	4.4	17.7	75.7	1.3	2.4	5.4	90.9
	Secondary	1,8	3.5	14.8	80.0	1.7	2.9	7.3	88.1
	Higher	1.1	3.0	7.2	88.6	1.2	1.8	7.9	89.1
		?^2^ = 75.7***				?^2^ = 29.4**			
Parity	0	2.3	5.0	16.2	76.4	2.6	4.4	11.6	81.4
	1	1.6	3.3	12.6	82.5	1.6	3.0	7.2	88.2
	2	0.7	2.2	11.4	85.7	1.6	2.0	5.8	90.6
	3	0.9	5.0	9.1	85.1	0.9	1.3	6.5	91.3
	4	1.2	3.5	11.9	83.4	1.6	2.4	4.6	91.4
		?^2^ = 47.7***				?^2^ = 71.8***			
Wealth Quintile	1. Poorest 20%	2.7	6.6	21.6	69.1	3.1	3.6	11.7	81.7
	2.	2.4	3.3	17.1	77.2	3.0	2.7	6.6	87.7
	3.	1.8	5.5	14.6	78.0	1.7	3.5	6.3	88.6
	4.	1.4	3.4	11.6	83.5	0.8	2.8	7.5	88.8
	5.Richest 20%	0.8	1.7	7.2	90.4	1.0	1.2	5.0	92.9
		?^2^ = 141.4**				?^2^ = 78.4***			
Do you listen radio at least once a week?	Yes	1,6	4.4	13.0	81.0	1.5	2.8	6.7	88.9
	No	1.8	2.8	17.2	78.2	2.2	2.2	8.6	87.1
		?^2^ = 11.9 **				?^2^ = 8.3**			
Do you watch TV atleast once a week?	Yes	1.6	3.7	12.6	82.2	1.3	2.3	6.3	90.1
	No	1.9	5.0	16.5	76.6	2.4	3.4	8.9	85.4
		?^2^ = 17.2**				?^2^ = 25.2***			
N		70	167	562	3296	60	88	236	3411
**Total (%)**		**1.7**	**4.1**	**13.7**	**80.5**	**1.6**	**2.3**	**6.2**	**89.9**

Significance levels: ***p<0.001; **p<0.05; *p<0.10.

#### Respondent’s age

The results presented in Table 1 indicate that both adult men and women who were aged 20–24 years were associated with high prevalence of underweight. About 23.7% of the male respondents aged 20–24 years compared to 15.6% of those aged 45–49 years were underweight. For the adult female population, approximately 17.6% of female respondents aged 20–24 years compared to 6.7% of those aged 45–49 years were underweight. Thus young age was associated with low BMI among the adult male and female population.

#### Place of residence

About a quarter of male respondents living in rural areas had low BMI compared to 14.7% of those living in cities/towns. Among women, 12.9% living in rural areas compared to 9.6% living in cities/towns were underweight. Therefore generally, adult population living in rural areas portrayed a high prevalence of low BMI than those residing in urban areas.

#### Marital status

Approximately 13.9% of currently married men had low BMI compared to 24.3% of currently married women. About 24.3% of never married men were underweight compared to 14.1% of never married women. As such low BMI was more prevalent among never married adult population.

#### Educational level

Approximately a quarter of men with no education compared to 11.4% of men with post-secondary education were underweight. Among women 17.9% with no education compared to 10.9% with post-secondary education were underweight. Twenty percent of men with secondary education compared to 11.9% of women with secondary education were underweight. Low BMI was more prevalent among adults with no education compared to those with higher levels of educational attainment.

#### Parity

Zero parity adult men and women compared to those with at least 4 children had higher percentages of low BMI, 23.5% versus 16.6% of men and 18.6% versus 8.6% of women. Overall low parity was associated with low BMI.

#### Socio-economic status as measured by wealth index

The proportion of adult men and women lives in the poorest 20% of the households had a higher prevalence of low BMI compared to those lives in the richest 20%. For instance, 30.9% of the undernourished adult male population belonged to the poorest 20% of households compared to 9.6% belonging to the richest 20% of households, while for undernourished adult women 18.4% belonged to the poorest 20% of the households compared to 11.1% belonging to the richest 20%. Therefore, it is clear that most of the adult population with low BMI was concentrated in the poorest 20% of the households. As such the socio-economic inequality determines whether one experiences low BMI or not.

#### Radio listening or television watching

About 21.8% of men who did not listen to a radio last week compared to 19.0% were shown to have low BMI or underweight. Approximately 12.9% of women who did not listen to a radio last week compared to 11.1% were shown to have low BMI or underweight. About 23.4% compared to 17.9% of men who did not watch a television last week were shown to be underweight. With regards to women approximately 14.6% compared to 19.9% of women who did not watch a television last week were shown to be underweight. Overall, a higher proportion of adult men and women who never listened to a radio or watched TV at least once a week had a high prevalence of under-nutrition.

### Determinants of under-nutrition

Table 2 shows multivariable logistic regression results with odds ratios and their standard errors for males and females, respectively. Never married men were 1.6 times more likely to be undernourished compared with those who were currently married or were in union (see Table 2). Men who had no education were 1.4 times more likely to be undernourished compared with those who had post-secondary education. Men who were in the poorest 20 percentile of household were 3.5 times more likely to be undernourished compared with those who were in the richest 20 percentile. All these relationships were statistically significant. Therefore men who were never married, had no education and were in the poorest household were more likely to experience low BMI.

**Table 2:** Predictors of under-nutrition among adult population aged 20–49 years, Botswana.

Factors		Men (n = 3419)		Female (n = 2188)	
		Odds ratio	S.E.	Odds ratio	S.E.
**Age**	20–24	1.00	0.22	2.40**	0.26
	25.29	0.99	0.21	2.05**	0.25
	30–34	1.08	0.21	1.67	0.25
	35–39	1.03	0.21	1.22	0.26
	40–44	1.02	0.22	1.05	0.27
	45–49 (*Ref*)	1.00	-	1.00	-
**Place of residence**	City/town (*Ref*)	1.00	-	1.00	-
	Urban village	1.13	0.11	1.19	0.12
	Rural	1.10	0.12	0.98	0.14
**Marital status**	Currently married/in union (*Ref*)	1.00	-	1.00	-
	Formerly married/in union	0.71	0.54	0.56	0.37
	Never married/in union	1.61***	0.11	1.15	0.10
**Educational level**	No education	1.45**	0.18	1.57**	
	Primary	1.66**	0.15	0.79	
	Secondary	1.31**	0.13	0.79	
	Higher (*Ref*)	1.00	-	1.00	
**Parity**	0 (*Ref*)	1.00	-	1.00	-
	1	0.85	0.12	0.61***	0.13
	2	0.76*	0.15	0.54***	0.15
	3	0.75	0.19	0.51***	0.19
	4+	0.75	0.18	0.49***	0.20
**Wealth Quintile**	1. Poorest 20%	3.46***	0.18	3.07***	0.21
	2.	2.30***	0.18	1.81**	0.21
	3.	2.23***	0.15	1.69**	0.17
	4.	1.59**	0.14	1.62**	0.15
	5. Richest 20% (*Ref*)	1.00	-	1.00	-
**Do you listen to radio** **at least once a week?**	Yes (*Ref*)	1.00	-	1.00	-
	No	0.88	0.11	0.88	0.12
**Do you watch TV at** **least once a week?**	Yes (*Ref*)	1.00	-	1.00	-
	No	0.84	0.11	1.37**	0.12

Significance levels: ***p<0.001; **p<0.05; *p<0.10; S.E. represents standard error.

Regarding women, younger women aged 20–24 years were 2.4 times more likely to be underweight compared to those aged 45–49 years. Women who had no education were 1.6 times more likely to be underweight compared with those who had post-secondary education. Women who were in the poorest 20 percentile of household were 3.1 times more likely to be undernourished compared with those who were in the richest 20 percentile. Women who did not watch TV at least once a week were 1.4 times more likely to be underweight compared to those who watched TV. Women of high parity were less likely to experience low BMI. All these relationships were statistically significant. Statistically significant predictors of underweight among adult females were being young, having no education, being in the poorest 20 percentile, and not having watched TV at least once a week.

## Discussion

Recently many developing countries have entered a nutrition transition in which rapid changes in food availability and physical activity patterns have led to an upward shift in BMI [31]. This transition has also been observed in countries in Latin America, Asia and Africa. The problem of underweight is also accompanied by increasing proportions of overweight and obese populations in Botswana comprising dual burden of health care [31].

Botswana is undergoing numerous changes, including demographic, life style and food habits, and socio-economic changes. Despite lack of historical data, the nutrition transition in Botswana has been explained by changes in lifestyle borrowed from Western countries of diets high in refined carbohydrates, saturated fats and sugars [32]. Recently, Botswana has seen an upsurge in the food availability and little is known on whether it benefitted all sections of the society to improve the nutritional level.

The prevalence of under nutrition was high among males than females. This is in contrast with the patterns observed in South Asia. The other main findings of this study were that both adult men and women who had no education and being in the poorest 20 percentile of the household were statistically significant predictors of under-nutrition. Among adult males, being never married was an additional predictor of under-nutrition. In addition to the above common factors, adult women who were young and lacked of access to mass media were at a greater risk for under-nutrition. Radio and TV are the key sources of information on various issues such as health. Through a radio or a television people receive and learn messages of healthy eating behaviours and lifestyles. As such those who own any of these media are expected to better informed and therefore be able to adopt a healthier lifestyle. The assumption in this study is that respondents without any of these do not have the knowledge on healthy eating behaviours and lifestyle and therefore are more likely to be underweight.

To our knowledge, this is the first study to document the prevalence and determinants of under-nutrition among adults aged 20–49 years in Botswana. The prevalence of under-nutrition was much more pronounced among men than women (19.5% and 10.1% respectively) and predictors of underweight were significantly associated with low socio-economic status (30.1% of the poorest adults vs. 9.6% of the richest adults). Because the prevalence of under-nutrition varies according to sex and socioeconomic groups, policies aimed at reducing under-nutrition among disadvantaged groups should be considered. These findings may provide important baseline data for assessing trends and potential predictors of under-nutrition for implementing programmes aimed at improving nutritional status and health of the population.

The study contributes significantly in understanding the prevalence of under-nutrition in Botswana and factor associated with it. Policy makers and programme managers can use the findings of this study to make appropriate interventions aimed at improving nutritional status of the population.

### Limitations of this study

The nature of relationship between infection and nutrition is well known and infection reduces the nutrient absorption capacity. Due to non-availability of information on HIV infection status from the survey, the study was not able to include indicators for infection or disease, for example being HIV positive, in the model. Examining the differences in consumption of food basket between poor and non-poor or male and female may also reveal possible reasons for differential nutritional outcome among them. The survey does not provide this information and is also beyond the scope of the paper.
